# Supplementary material for: Metabolite Fingerprinting for Identification of Panax ginseng Metabolites Using Internal Extractive Electrospray Ionization Mass Spectrometry
Source: Foods. 2023 Mar 9;12(6):1152. doi: 10.3390/foods12061152 (PMC10048038; doi:10.3390/foods12061152)
Supplement: Supplementary file 1 [file foods-12-01152-s001.zip › foods-2071348-supplementary.pdf]

Supplementary information

Differentiation of different species of ginseng by internal extraction electrospray ionization mass spectrometry

Xueyan Yuan[1], Xiaoping Zhang[1], Jiaquan Xu[1], Jianhua Ye[2], Zhendong Yu[1], and Xinglei Zhang[1,*]

[1] Jiangxi Key Laboratory for Mass Spectrometry and Instrumentation, East China Institute of Technology, Nanchang 330013, P. R. China

[2] School of Nuclear Science and Engineering, East China University of Technology, East China University of Technology, Nanchang, Jiangxi, 330013. China

*Corresponding author:

Dr. Xinglei Zhang

leizi8586@126.com

Table of Contents

Page

Table S1. The structure of ginsenosides 3-4

Figure S1. Effect of mass spectrometry parameters on the signal intensity of three representative ginsenosides 4

Figure S2. The conventional cleavage of ginsenosides 5

Figure S3. Tandem MS analysis of chlorination peaks of ginsenosides detected by iEESI-MS 5

Figure S4. Fingerprint spectra of ginsenoside standard samples Rb1, Re, and Ro under negative and positive ion modes. 6

Figure S5. Fingerprint spectra of the ginseng under forest samples with four different solvents. 6

Table S1. The structure of ginsenosides

No.	Structure	Ginsenoside	R1	R2	References
1		Rg3	-Glc^{2}-^{1}Glc	-H	[1]
2		Rs3	-Glc^{2}-^{1}Glc^{6}-Ac	-H	[2]
3		Rd	-Glc^{2}-^{1}Glc	-Glc	[3]
4	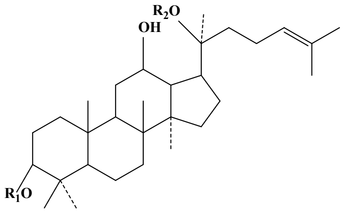	Pseudoginsenoside-Rc1	-Glc^{2}-^{1}Glc^{6}-Ac	-Glc	[4]
5		Rc	-Glc^{2}-^{1}Glc	-Glc^{6}-^{1}Ara(f)	[3]
6		Rb2	-Glc^{2}-^{1}Glc	-Glc^{6}-^{1}Ara(p)	[3]
7		Rb3	-Glc^{2}-^{1}Glc	-Glc^{6}-^{1}Xyl	[3]
8	(20S)-Protopanaxadiol(R_1=R_2=H)	Rb1	-Glc^{2}-^{1}Glc	-Glc^{6}-^{1}Glc	[3]
9		Rs1	-Glc^{2}-^{1}Glc^{6}-Ac	-Glc^{6}-^{1}Xyl	[2]
10		Rs2	-Glc^{2}-^{1}Glc^{6}-Ac	-Glc^{6}-^{1}Ara(f)	[2]
11		Quinquenoside-R1	-Glc^{2}-^{1} Glc^{6}-Ac	-Glc^{6}-^{1}Glc	[5]

No.	Structure	Ginsenoside	R1	R2	References
12		Rh1	-Glc	-H	[6]
13		Notoginsenoside-R2	-Glc^{2}-^{1}Xyl	-H	[3]
14		Rg2	Glc^{2}-^{1}Rha	-H	[1]
15		Rg1	-Glc	-Glc	[1]
16		Rf	-Glc^{2}-^{1}Glc	-H	[3]
17		Ma-Rg1	-Glc^{6}-Malonyl	-Glc	[3]
19	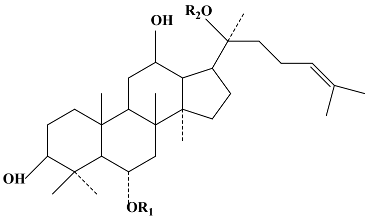	Notoginsenoside-R1	-Glc^{2}-^{1}Xyl	-Glc	[2]
20		Re	Glc^{6}-^{1}Rha	-Glc	[3]
21		20-glu-Rf	-Glc^{2}-^{1}Glc	-Glc	[3]
22	(20S）Protopanaxatriol(R1=R2 =H)	Notoginsenoside-N	-Glc^{4}-^{1}Glc	-Glc	[7]
23		Re1	-Glc	-Glc^{3}-^{1}Glc	[7]
24		Re2	-Glc^{3}-^{1}Glc	-Glc	[7]
25		Re3	-Glc	-Glc^{4}-^{1}Glc	[7]
29		Notoginsenoside-R3	-Glc	-Glc^{6}-^{1}Glc	[8]

No.	Structure	Ginsenoside	R1	R2	References
26		Chikusetsusaponin-Iva	-GlcA	-Glc	[3]
27		Zingibroside-R1	-GlcA^{2}-^{1}Glc	-H	[3]

28	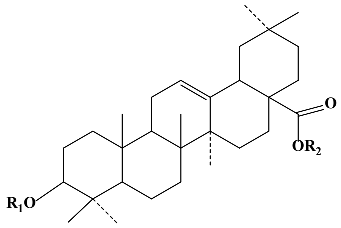	R0	-GlcA^{2}-^{1}Glc	-Glc	[3]

No.	Structure	Ginsenoside	R1	R2	References
30	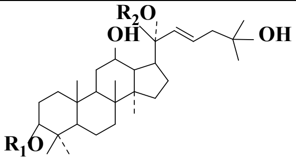	Korean−R2	-Glc^{2}-^{1}Glc	Glc^{6}-^{1}Glc	[9]

No.	Structure	Ginsenoside	R1	R2	References
31	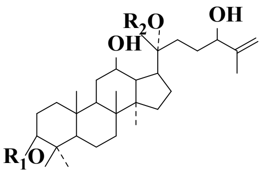	V	-Glc^{2}-^{1}Glc	Glc^{6}-^{1}Glc	[10]

No.	Structure	Ginsenoside	R1	R2	References
32	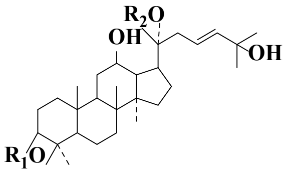	Notoginsenoside-A	-Glc^{2}-^{1}Glc	Glc^{6}-^{1}Glc	[8]

Note: Ma-Rf, Ma-(20-glu-Rf), Ma-Notoginsenoside-N, Ma-Re1, Ma-Re2, Ma-Re3 are the structures of Rf, 20-glu-Rf, Notoginsenoside-N, Re1, Re2 and Re3 with a Ma group added to them respectively(Ma: Malonyl)

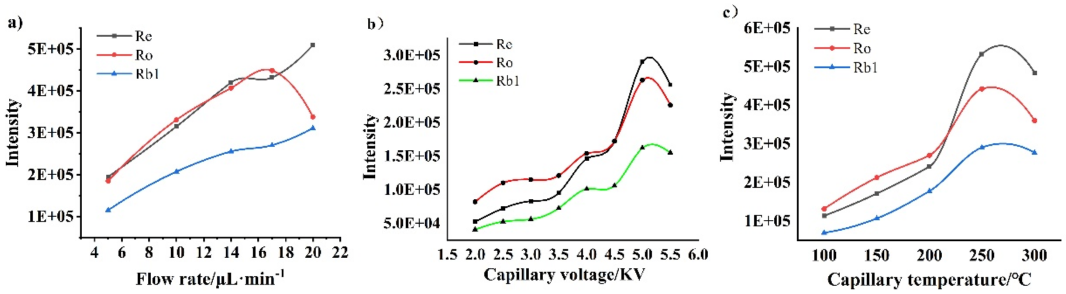

Figure S1. Effect of mass spectrometry parameters on the signal intensity of three representative ginsenosides. (a) Effect of extractant flow rate on the signal intensity of ginsenosides, (b) Effect of capillary voltage on the signal intensity of ginsenosides, (c) Effect of capillary temperature on the signal intensity of ginsenosides.

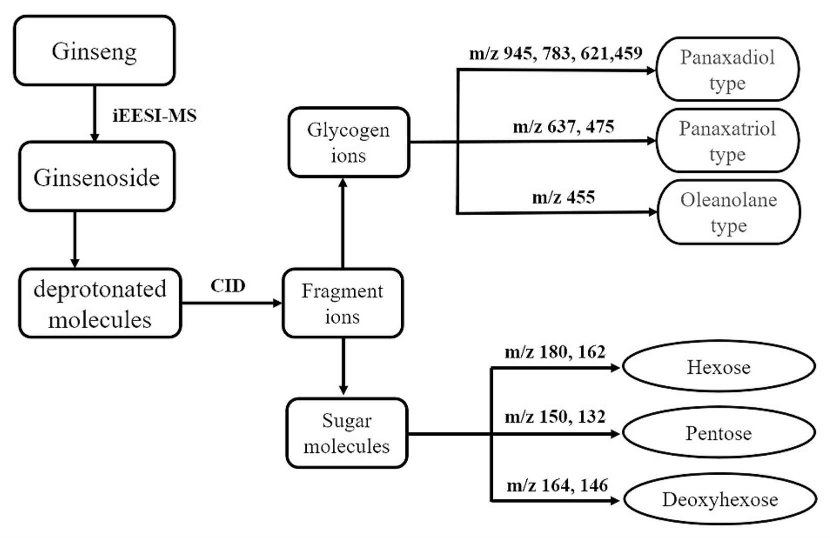

Figure S2. The conventional cleavage of ginsenosides.

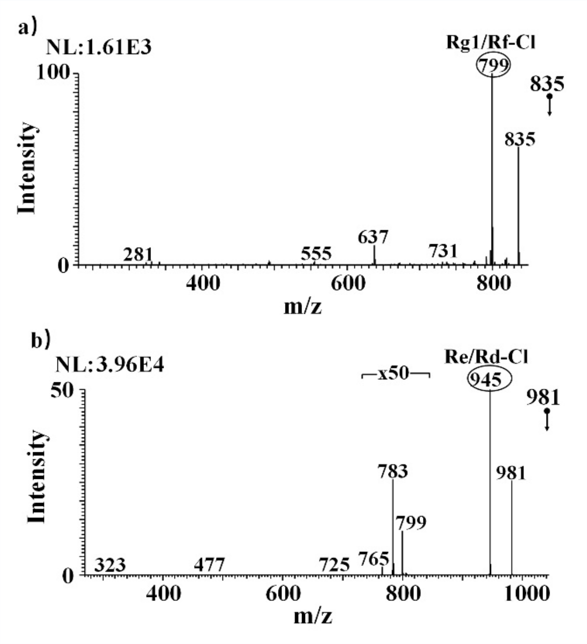

Figure S3. Tandem MS analysis of chlorination peaks of ginsenosides detected by iEESI-MS. (a) MS^2 spectrum of *m/z* 835→, (b) MS^2 spectrum of *m/z* 981→.

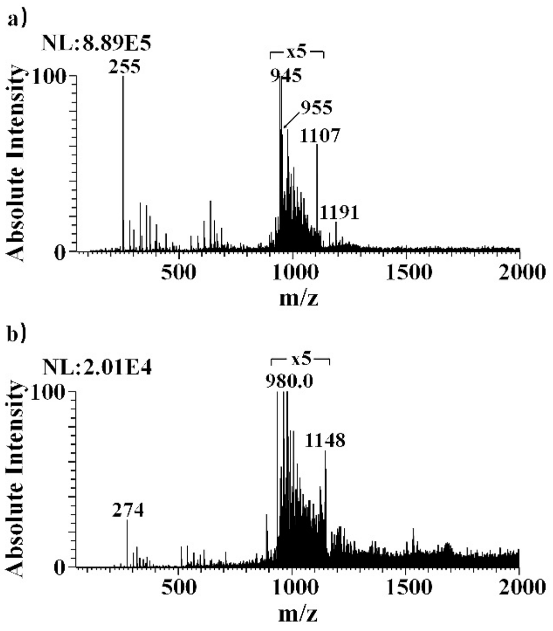

Figure S4. Fingerprint spectra of ginsenoside standard samples Rb1, Re, and Ro under negative and positive ion modes. (a) Fingerprint spectra of ginsenosides Rb1, Re, and Ro in negative ion mode, (b) Fingerprint spectra of ginsenosides Rb1, Re, and Ro in positive ion mode.

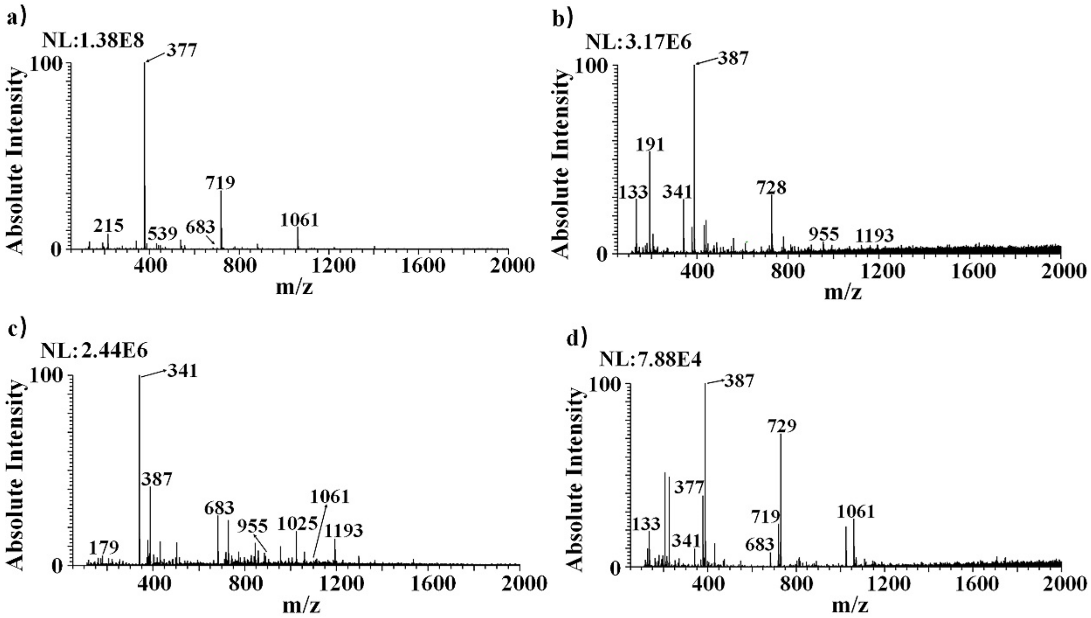

Figure S5. Fingerprint spectra of the ginseng under forest samples with four different solvents. (a) Fingerprint spectra of the ginseng under forest samples with 0.5 mM ammonium chloride in methanol solvent, (b) Fingerprint spectra of the ginseng under forest samples with 0.1% formic acid in water/ethanol (v:v=1:1), (c) Fingerprint spectra of the ginseng under forest samples with 10 mM ammonium acetate in acetonitrile/methanol (v:v=1:1), (d) Fingerprint spectra of the ginseng under forest samples with 0.1% formic acid in acetonitrile/ethanol (v:v=1:1).

References

1. Fuzzati, N.; Gabetta, B.; Jayakar, K.; Pace, R.; Peterlongo, F. Liquid chromatography–electrospray mass spectrometric identification of ginsenosides in Panax ginseng roots. *J Chromatogr A.* **1999**, 854, 69-79.
2. Wu, W.; Song, F.; Guo, D.; Mi, J.; Qin, Q.; Yu, Q.; Liu, S. Mass Spectrometry-Based Approach in Ginseng Research: A Promising Way to Metabolomics. *CURR ANAL CHEM.* **2012**, 8, 43-66.
3. Xie, Y.Y.; Luo, D.; Cheng, Y.J.; Ma, J.F.; Wang, Y.M.; Liang, Q.L.; Luo, G.A. Steaming-induced chemical transformations and holistic quality assessment of red ginseng derived from Panax ginseng by means of HPLC-ESI-MS/MS(n)-based multicomponent quantification fingerprint. *J. Agric. Food Chem.* **2012**, 60, 8213-8224.
4. Zhu, G.Y.; Li, Y.W.; Hau, D.K.; Jiang, Z.H.; Yu, Z.L.; Fong, W.F. Acylated protopanaxadiol-type ginsenosides from the root of Panax ginseng. *Chem Biodivers.* **2011**, 8, 1853-1863.
5. Lin, H.; Zhu, H.; Tan, J.; Wang, C.; Dong, Q.; Wu, F.; Wang, H.; Liu, J.; Li, P.; Liu, J. Comprehensive Investigation on Metabolites of Wild-Simulated American Ginseng Root Based on Ultra-High-Performance Liquid Chromatography-Quadrupole Time-of-Flight Mass Spectrometry. *J. Agric. Food Chem.* **2019**, 67, 5801-5819.
6. Tam, D.N.H.; Truong, D.H.; Nguyen, T.T.H.; Quynh, L.N.; Tran, L.; Nguyen, H.D.; Shamandy, B.E.; Le, T.M.H.; Tran, D.K.; Sayed, D.; Vu, V.V.; Mizukami, S.; Hirayama, K.; Huy, N.T. Ginsenoside Rh1: A Systematic Review of Its Pharmacological Properties. *Planta. Med.* **2018**, 84, 139-152.
7. Zhu, G.Y.; Li, Y.W.; Hau, D.K.; Jiang, Z.H.; Yu, Z.L.; Fong, W.F. Protopanaxatriol-type ginsenosides from the root of Panax ginseng. *J. Agric. Food Chem.* **2011**, 59, 200-205.
8. Yoshikawa, M.; Murakami, T.; Ueno, T.; Yashiro, K.; Hirokawa, N.; Murakami, N.; Yamahara, J.; Matsuda, H.; Saijoh, R.; Tanaka, O. Bioactive saponins and glycosides. VIII. Notoginseng (1): new dammarane-type triterpene oligoglycosides, notoginsenosides-A, -B, -C, and -D, from the dried root of Panax notoginseng (Burk.) F.H. Chen. *Chem Pharm Bull.* **1997**, 45, 1039-1045.
9. Kim, D.S.; Chang, Y.J.; Zedk, U.; Zhao, P.; Liu, Y.Q.; Yang, C.R. Dammarane saponins from Panax ginseng. *Phytochemistry* **1995**, 40, 1493-1497.
10. Yang, W.Z.; Ye, M.; Qiao, X.; Liu, C.F.; Miao, W.J.; Bo, T.; Tao, H.Y.; Guo, D.A. A strategy for efficient discovery of new natural compounds by integrating orthogonal column chromatography and liquid chromatography/mass spectrometry analysis: Its application in Panax ginseng, Panax quinquefolium and Panax notoginseng to characterize 437 potential new ginsenosides. *Anal. Chim. Acta.* **2012**, 739, 56-66.
